# Social and Emotional Wellbeing of Aboriginal Community Controlled Health Services Staff during the COVID-19 Pandemic

**DOI:** 10.3390/ijerph20126060

**Published:** 2023-06-06

**Authors:** Smriti Nepal, Sandra Bailey, Jamie Newman, Lachlan Wright, Natalie Smith, Michelle Dickson, Anna Williamson

**Affiliations:** 1 Sax Institute, Glebe, NSW 2037, Australia; sandra.bailey@saxinstitute.org.au; 2 Orange Aboriginal Medical Service, Orange, NSW 2800, Australia; ceo@oams.net.au; 3 Tharawal Aboriginal Corporation, Airds, NSW 2560, Australia; lachlan.wright@tacams.com.au; 4 Riverina Medical and Dental Aboriginal Corporation, Wagga Wagga, NSW 2650, Australia; natalie.smith@rivmed.org.au; 5 Sydney School of Public Health, University of Sydney, Sydney, NSW 2006, Australia; michelle.dickson@sydney.edu.au; 6 Centre for Evidence and Implementation, Carlton, VIC 3053, Australia; anna.williamson@ceiglobal.org

**Keywords:** healthcare workers, social and emotional wellbeing, Aboriginal and Torres Strait Islander health, primary healthcare

## Abstract

This study explores the impact of the COVID-19 pandemic on the work and social and emotional well-being (SEWB) of staff at Aboriginal Community Controlled Health Services (ACCHS) in Australia. Between September and November 2021, staff from three ACCHSs in New South Wales completed an online survey to report changes to their roles, concerns about becoming infected with the COVID-19 virus, and job satisfaction in the last month. The survey measured emotional exhaustion and psychological distress by using the Maslach Burnout Inventory-Human Services Survey and Kessler-5 scale, respectively. The survey determined staff’s access to SEWB support. Descriptive statistics were calculated for each variable. Among 92 staff from three ACCHSs, 36% reported a COVID-19-related change in their role and 64% were concerned about becoming infected. In spite of the pandemic, most staff (69%) were satisfied with their job. While most staff were not burnt out or psychologically distressed, 25% had high emotional exhaustion and 30% had high to very high psychological distress. Relatedly, 37% had accessed SEWB support at least once in their lifetime and 24% had accessed support in the last month. As the pandemic continues, it is important to identify factors influencing burnout or psychological distress among ACCHS staff and implement evidence-based solutions.

## 1. Introduction

Globally, Indigenous peoples experience a disproportionately high burden of disease and poor health relative to their non-Indigenous counterparts [1]. The health gap is exacerbated by barriers that they encounter in accessing mainstream healthcare services. Barriers include but are not limited to discrimination, sense of alienation, cost, communication difficulties, location, and lack of cultural safety [2,3]. Indigenous primary healthcare (PHC) services can fill the gap and impact health outcomes by improving accessibility, fostering community participation, cultural safety, and offering comprehensive healthcare [4]. Additionally, Indigenous PHC services are likely to improve health outcomes because they are community-controlled and are underpinned by community values [4,5]. In Australia, the comprehensive PHC services, i.e., Aboriginal Community Controlled Health Services (ACCHSs), were initiated (in 1971) by and for Aboriginal communities, to improve Aboriginal and Torres Strait Islander health by overcoming barriers and providing culturally responsive, holistic health care [6].

There are currently 144 ACCHSs across Australia employing 6000 staff, 3500 of whom are Aboriginal and/or Torres Strait Islander [7]. ACCHSs increase Aboriginal and Torres Strait Islander people’s access to primary healthcare, and improve outcomes for maternal and child health, chronic disease, sexual health, and mental health [8]. Aboriginal and Torres Strait Islander people report positive healthcare experiences at ACCHSs due to culturally appropriate care, feeling accepted and understood, and the culturally safe setting [8]. ACCHS staff’s role, particularly the Aboriginal and Torres Strait Islander staff, is not limited to providing clinical services. They are relied on by the communities to provide support or counselling, often out of working hours [9]. Where there is a scarcity of clinicians, non-clinical staff may have to perform medical procedures (e.g., health checks, vaccinations, PAP smear) that are carried out by clinicians in mainstream services [10]. ACCHS staff can help improve patient attendance and treatment completion, continuity of care, and reduce discharge against medical advice [11,12].

During the coronavirus (COVID-19) pandemic, ACCHSs have taken on new roles, acting as temporary testing and vaccination sites, educating communities about COVID-19 safety behaviour, and supporting older community members to self-isolate [13,14]. ACCHS staff are also called on to offer COVID-19-related informal counselling and education to local communities [14]. Increased demands on healthcare services are associated with negative impacts on the social and emotional wellbeing (SEWB) of healthcare workers (HCWs) [15]. Studies show that during the COVID-19 pandemic, mental health symptoms such as burnout, anxiety, depression, and psychological distress have been common among HCWs in all levels of healthcare (primary, secondary, tertiary) [16,17,18]. Leaders at partner ACCHSs have noted that COVID-19-related service-level changes, heightened community distress, and threats to one’s own health have been taxing the SEWB of staff. They have observed decreased staff morale and increased stress and absenteeism among staff. Despite the increased workload and high risk of mental health symptoms, there is limited empirical evidence on the SEWB of HCWs working with Aboriginal and Torres Strait Islander people.

The current study examines the experience of ACCHS staff at three services in New South Wales (NSW), Australia during the COVID-19 pandemic, specifically following the Delta variant outbreak and coinciding with the peak of vaccine rollout. The study aims to identify staff’s perception about changes in their role, levels of concern about infection, perceptions of community distress, and levels of work-related emotional exhaustion and psychological distress.

## 2. Materials and Methods

### 2.1. Locations and Participants

Three ACCHSs participated in the study, one was located in urban and two in regional NSW. The Chief Executive Officers at the ACCHSs had identified the increased risk that the COVID-19 pandemic posed on the SEWB of their staff, making them a priority for urgent support.

Staff employed at the participating ACCHSs were eligible for the study if they were: ≥18 years old, fluent in English, and provided written consent to participate. Any staff member (for example, clinical, non-clinical, management, administrative, support) could complete the survey, there was no restriction based on staff’s duration of employment at ACCHS, or hours worked per week.

### 2.2. Data Collection

An online survey was administered through REDCap, with the link and participant information materials emailed to all staff by Chief Executive Officers or Aboriginal Research Officers at the participating ACCHSs. The survey was open for three weeks, and staff were sent reminder to complete the survey at the start of the week. Data collection took place between September and November 2021. During this time period, Australia was emerging from a difficult period of dealing with the Delta variant outbreak (June 2021 onwards) [19] and the rapid increase in vaccination rate in Australia (July 2021 onwards) [20]. Like mainstream health workers, ACCHS staff were learning about the vaccine while vaccinating communities and working to overcome vaccine hesitancy all at the same time.

### 2.3. Measures

The survey was developed in consultation with ACCHS leaders. It included questions on demographic characteristics (gender, age group, Aboriginality, relationship status, role at ACCHS), staff’s perceptions of how the COVID-19 pandemic impacted services, and any change in their roles. It also included questions to determine concern about COVID-19 infection, perceived community distress, job satisfaction, levels of emotional exhaustion and psychological distress, and SEWB help-seeking. The questions required participants to reflect on their experiences in the four weeks prior to the survey.

The Emotional Exhaustion subscale from the Maslach Burnout Inventory-Human Services Survey (MBI-HSS) was used to measure work-related emotional exhaustion [21]. Using nine items, the subscale measures the emotional response to being overextended by work. Items were scored on a seven-point scale, from zero (never) to six (all the time). Scores can range from 0 to 54 with 0–16 indicating low emotional exhaustion, 17–26 indicating moderate, and ≥27 indicating high emotional exhaustion [21]. Despite overlaps in the symptoms of emotional exhaustion and psychological distress, MBI-HSS specifically only measures work-related stressors.

The Kessler-5 (K-5) scale was used to measure psychological distress. The scale has been validated for Aboriginal and Torres Strait Islander people and comprises a subset of five questions from the Kessler-10 [22]. It measures negative emotional states experienced by participants in the last month. Each item is scored on a five-point scale, from one (none of the time) to five (all of the time). Scores between 5 and 11 indicate low-moderate and those between 12 and 25 indicate high to very high psychological distress [22].

### 2.4. Statistical Analysis

Frequencies and percentages were calculated for all variables. For MBI-HSS, the percentage of participants with low, moderate, or high levels of emotional exhaustion was calculated. Similarly, for the K-5 scale, the percentage of participants with low, moderate, high, and very high psychological distress was calculated. Stata 17 was used for data analysis.

### 2.5. Ethics Approval

Ethics approval was obtained from the University of New South Wales (HC210540) and Aboriginal Health and Medical Research Council of NSW (1834/21) ethics committees.

## 3. Results

### 3.1. Demographic Characteristics

Ninety-two staff from three ACCHSs completed the survey, the response rate was approximately 44%. Most participants (64%) were based at the two regional ACCHSs. Most of the respondents identified as Aboriginal (57%) or female (77%) (Table 1). Twenty-seven percent of the respondents were between 30 and 39 years, 34% were married, and 18% were in a de facto relationship. Thirty-three percent of the respondents worked as clinical staff (doctors, nurses, dentists, physiotherapists, psychologists, Aboriginal Health Workers). The majority (73%) had worked full-time (40 h per week) in the last month. Thirty-seven percent had worked at the ACCHS for 1–3 years and 61% had started working the ACCHS before the COVID-19 pandemic started. At the time of the survey, 98% of the respondents had received two doses of the COVID-19 vaccine.

### 3.2. Change in Role Due to the Pandemic

Thirty-six percent of the staff reported COVID-19-related changes to their roles in the last month. Half of those who reported changes performed additional tasks that were normally not part of their role, and 45% could not perform all or a part of their usual roles. For example, staff reported reduced community engagement, started screening patients (via telephone, telehealth, at the car park) to ascertain the requirement of in-person consultation, and distributed food hampers to isolated community members.

### 3.3. Concern about COVID-19 Infection

In the last month, 64% of the respondents were concerned about becoming infected with the COVID-19 virus. Common reasons for concern were the risk of transmission to family (27%) and the community (24%), being unwell (20%), isolation (18%), and the effect on employment status (8%). Staff were also concerned about the effect of the pandemic on the healthcare system, long-term health impacts, and being stigmatized against (in case of infection), and pregnant staff were concerned about the effect of infection on their foetus.

### 3.4. Community Distress

Seventy-one percent of the ACCHS staff identified increased distress among their clients in the last month, compared to the pre-COVID-19 period. The most common reasons for community distress were fear of isolation from family and friends (59%), concern about becoming infected with COVID-19 (53%), concern about infecting family (49%), and uncertainty about the pandemic (49%). Staff reported that the increase in distress among clients did not impact their work performance.

### 3.5. Job Satisfaction

Despite the COVID-19 pandemic, ACCHS staff were either very satisfied (38%) or satisfied (31%) with their job. Among those who were not satisfied (10%), the common reasons for dissatisfaction were upheaval at their ACCHS, increased workload, and inability to enjoy work.

### 3.6. Work-Related Emotional Exhaustion

The mean Emotional Exhaustion score was 19.77, indicating a moderate level of emotional exhaustion among ACCHS staff in the last month. Approximately half (46%) of the staff had low and 25% had high emotional exhaustion.

### 3.7. Psychological Distress

The mean K-5 score was 9.59, indicating an overall low-moderate level of psychological distress among ACCHS staff in the last month. Seventy percent of participants had low-moderate, and 30% had high to very high levels of psychological distress.

### 3.8. SEWB Support

Among the 92 ACCHS staff, 37% had accessed SEWB support at least once in their lifetime, and 24% in the last month. Most of the staff who sought SEWB support (64%) went to mental health professionals (for example, mental health nurses, counsellors, social workers, and occupational therapists), and the majority (86%) of those who accessed support were satisfied with the services they received. The most common reason for staff not accessing support was thinking they did not require SEWB support (67%).

## 4. Discussion

To our knowledge, this is the first study to explore the SEWB needs of ACCHS staff during the COVID-19 pandemic. More than one-third of ACCHS staff reported COVID-19-related changes to their role. Most staff were concerned about becoming infected with the COVID-19 virus and noted that community distress was high. The majority of staff in the study reported high job satisfaction and had low levels of emotional exhaustion and psychological distress. Twenty-five percent of the staff had high emotional exhaustion and 30% had high to very high psychological distress. Relatedly, 37% of all respondents had accessed SEWB support at least once in their lifetime and 24% sought support in the past month.

Throughout the COVID-19 pandemic, ACCHS staff have performed tasks that are not part of their usual role, and the resultant change in their roles have led to reduced community engagement; these factors are likely to have contributed to emotional exhaustion [15]. Some staff members in the study belong to the communities they serve and, during the pandemic, they have often been called to provide counselling or support, even outside of work hours. This may have impacted their ability to ‘switch off’ and relax. The increased workload and inability to separate work from personal life could also have contributed to burnout and psychological distress [23,24]. Despite changes in their role, and levels of emotional exhaustion and psychological distress, most ACCHS staff were satisfied with work. This could be due to support from colleagues, positive mentorship, culturally safe workspaces, clinical and cultural supervision, and the ability to make a difference in their communities [25].

While ACCHS staff in the current study have demonstrated great resilience in the face of the increased demands, almost one-third are emotionally exhausted or psychologically distressed. The findings from the study are comparable to other Australian and international studies [26,27]. In a rapid review of the international literature, the prevalence of burnout among HCWs was 22–46%, anxiety was 7–97%, depression was 11–62%, stress was 2–94%, and post-traumatic stress was 4–57% [26]. In a study of Australian HCWs, 71% suffered from burnout, 60% suffered from anxiety, and 57% from suffered from depression [27]. Similarly, in a longitudinal study of Australian primary HCWs, mental health symptoms worsened from March/April to September/October 2021. At the second time point (that partly overlapped with our study), 19% of HCWs suffered from anxiety, 31% from depression, and 26% from stress [18]. None of the studies focused specifically on HCWs working with Indigenous peoples who have poorer health outcomes compared to the general population.

Burnout and psychological distress in HCWs are linked to increased absenteeism rates and work-related errors, decreased job satisfaction, and poor patient outcomes [28]. As the pandemic continues, it is important that efforts are made to support the wellbeing of ACCHS staff. Management can help minimise staff’s concern about infection by equipping them with adequate personal protective equipment and medications, providing regular training in safety protocols and up-to-date COVID-19 management guidelines [25,29]. Well-being programs, psychological services (e.g., psychotherapy, counselling), and initiatives such as the Employee Assistance Program can be helpful in supporting HCWs’ wellbeing [29], but we were unable to identify evaluations of such programs or services designed to support the SEWB of ACCHS staff.

The findings from the study should be examined in light of its limitations. The cross-sectional design does not allow tracking changes in emotional exhaustion and psychological distress throughout the pandemic; further rounds of data collection are underway to facilitate this. While the K-5 questionnaire used in the study has been validated in Aboriginal and Torres Strait Islander population, the MBI-HSS questionnaire has not. However, it has been extensively adapted and validated in diverse population groups, in Australia and overseas [30]. Despite the limitations, as the first exploration of this topic, the findings can contribute towards Aboriginal health services planning to ensure improved workloads for ACCHS staff. The forthcoming longitudinal data on ACCHS staff SEWB and the planned evaluation of ACCHS-nominated strategies to support staff wellbeing will also add to the evidence base.

## 5. Conclusions

During the COVID-19 pandemic, ACCHSs have played a vital role in supporting Aboriginal and Torres Strait Islander communities, taking on critical additional functions and rapidly instituting major service-level changes. In spite of the challenges the changes have presented, most ACCHS staff in the current study reported job satisfaction and good SEWB. A significant minority of staff, however, were found to have high emotional exhaustion and psychological distress, suggesting that more support is needed for this important group of health service providers.

## Figures and Tables

**Table 1:** Demographic characteristics of participants.

Variable	Frequency (*N* = 92)	Percent (%)
*Gender*		
Female	71	77
Male	20	22
Prefer not to say	1	1
*Age group (years)*		
18 to 29	23	25
30 to 39	25	27
40 to 49	20	22
50 to 59	18	20
≥60	6	6
*Relationship*		
Single	34	37
De facto relationship or married	48	52
Separated	5	5
Prefer not to say	5	5
*Aboriginality*		
Aboriginal	52	57
Neither Aboriginal nor Torres Strait Islander	38	41
Prefer not to say	2	2
*Role at ACCHS*		
Clinical staff (e.g., doctor, dentist, nurse, physiotherapist, psychologist, Aboriginal Health Worker)	30	33
Non-clinical Aboriginal Health Worker (e.g., program manager, program coordinator, project officer)	12	13
Clinic Manager	3	3
Administrative staff (e.g., HR staff, Finance officers, receptionist)	21	23
Other staff (e.g., transport officer, social service providers such as NDIS workers, family preservation caseworkers etc)	26	28

## Data Availability

The data presented in this study are available on request from the corresponding author. The data are not publicly available to ensure confidentiality of the participants.

## References

[1] Anderson I., Robson B., Connolly M., Al-Yaman F., Bjertness E., King A., Tynan M., Madden R., Bang A., Coimbra C.E.A. (2016). Indigenous and tribal peoples’ health (The Lancet–Lowitja Institute Global Collaboration): A population study. Lancet.

[2] King M., Smith A., Gracey M. (2009). Indigenous health part 2: The underlying causes of the health gap. Lancet.

[3] Nolan-Isles D., Macniven R., Hunter K., Gwynn J., Lincoln M., Moir R., Dimitropoulos Y., Taylor D., Agius T., Finlayson H. (2021). Enablers and Barriers to Accessing Healthcare Services for Aboriginal People in New South Wales, Australia. Int. J. Environ. Res. Public Health.

[4] Harfield S.G., Davy C., McArthur A., Munn Z., Brown A., Brown N. (2018). Characteristics of Indigenous primary health care service delivery models: A systematic scoping review. Glob. Health.

[5] Halseth R., Murdock L. (2020). Supporting Indigenous Self-Determination in Health: Lessons Learned from a Review of Best Practices in Health Governance in Canada and Internationally.

[6] Khoury P. (2015). Beyond the biomedical paradigm: The formation and development of Indigenous community-controlled health organizations in Australia. Int. J. Health Serv..

[7] National Aboriginal Community Controlled Health Organisation (NACCHO) (2022). About NACCHO: National Aboriginal Community Controlled Health Organisation (NACCHO). https://www.naccho.org.au/about-us/.

[8] Campbell M.A., Hunt J., Scrimgeour D.J., Davey M., Jones V. (2017). Contribution of Aboriginal Community-Controlled Health Services to improving Aboriginal health: An evidence review. Aust. Health Rev..

[9] Topp S.M., Edelman A., Taylor S. (2018). “We are everything to everyone”: A systematic review of factors influencing the accountability relationships of Aboriginal and Torres Strait Islander health workers (AHWs) in the Australian health system. Int. J. Equity Health.

[10] Mitchell M., Hussey L.M. (2006). The Aboriginal health worker. Med. J. Aust..

[11] Stamp G., Champion S., Anderson G., Warren B., Stuart-Butler D., Doolan J., Boles C., Callaghan L., Foale A., Muyambi C. (2008). Aboriginal maternal and infant care workers: Partners in caring for Aboriginal mothers and babies. Rural Remote Health.

[12] Deroy S., Schütze H. (2019). Factors supporting retention of aboriginal health and wellbeing staff in Aboriginal health services: A comprehensive review of the literature. Int. J. Equity Health.

[13] Eades S., Eades F., McCaullay D., Nelson L., Phelan P., Stanley F. (2020). Australia’s First Nations’ response to the COVID-19 pandemic. Lancet.

[14] Crooks K., Casey D., Ward J.S. (2020). First Nations people leading the way in COVID-19 pandemic planning, response and management. Med. J. Aust..

[15] Chigwedere O.C., Sadath A., Kabir Z., Arensman E. (2021). The impact of epidemics and pandemics on the mental health of healthcare workers: A systematic review. Int. J. Environ. Res. Public Health.

[16] Vizheh M., Qorbani M., Arzaghi S.M., Muhidin S., Javanmard Z., Esmaeili M. (2020). The mental health of healthcare workers in the COVID-19 pandemic: A systematic review. J. Diabetes Metab. Disord..

[17] Sirois F.M., Owens J. (2021). Factors associated with psychological distress in health-care workers during an infectious disease outbreak: A rapid systematic review of the evidence. Front. Psychiatry.

[18] Holton S., Wynter K., Peeters A., Georgalas A., Yeomanson A., Rasmussen B. (2023). Psychological wellbeing of Australian community health service staff during the COVID-19 pandemic: A longitudinal cohort study. BMC Health Serv. Res..

[19] Kapon A., Sheppeard V., Gonzalez N., Draper J., Zhu A., Browne M., Sullivan E., Mihajlovic M., Rockett R., Ferson M. (2021). Bondi and beyond. Lessons from three waves of COVID-19 from 2020. Public Health Res. Pract..

[20] Nicholas J., Evershed N., Ball A. (2022). COVID-19 Australia Data Tracker: Coronavirus Cases, Deaths, Hospitalisations and Vaccination. The Guardian.

[21] Maslach C., Jackson S.E., Leiter M.P. (1996). Maslach Burnout Inventory Manual.

[22] Australian Bureau of Statistics (2015). Australian Aboriginal and Torres Strait Islander Health Survey: Users’ Guide, 2012–2013: Australian Bureau of Statistics. https://www.abs.gov.au/ausstats/abs@.nsf/Lookup/4727.0.55.002Chapter3602012-13.

[23] Peiris D., Brown A., Howard M., Rickards B.A., Tonkin A., Ring I., Hayman N., Cass A. (2012). Building better systems of care for Aboriginal and Torres Strait Islander people: Findings from the Kanyini health systems assessment. BMC Health Serv. Res..

[24] Roberts R., Wong A., Jenkins S., Neher A., Sutton C., O’Meara P., Frost M., Bamberry L., Dwivedi A. (2021). Mental health and well-being impacts of COVID-19 on rural paramedics, police, community nurses and child protection workers. Aust. J. Rural Health.

[25] Lai G.C., Taylor E.V., Haigh M.M., Thompson S.C. (2018). Factors Affecting the Retention of Indigenous Australians in the Health Workforce: A Systematic Review. Int. J. Environ. Res. Public Health.

[26] Major A., Hlubocky F.J. (2021). Mental health of health care workers during the COVID-19 pandemic and evidence-based frameworks for mitigation: A rapid review. medRxiv.

[27] Smallwood N., Karimi L., Bismark M., Putland M., Johnson D., Dharmage S.C., Barson E., Atkin N., Long C., Ng I. (2021). High levels of psychosocial distress among Australian frontline healthcare workers during the COVID-19 pandemic: A cross-sectional survey. Gen. Psychiatry.

[28] De los Santos J.A.A., Labrague L.J. (2021). The impact of fear of COVID-19 on job stress, and turnover intentions of frontline nurses in the community: A cross-sectional study in the Philippines. Traumatology.

[29] Magnavita N., Chirico F., Garbarino S., Bragazzi N.L., Santacroce E., Zaffina S. (2021). SARS/MERS/SARS-CoV-2 outbreaks and burnout syndrome among healthcare workers. An umbrella systematic review. Int. J. Environ. Res. Public Health.

[30] Poghosyan L., Aiken L.H., Sloane D.M. (2009). Factor structure of the Maslach burnout inventory: An analysis of data from large scale cross-sectional surveys of nurses from eight countries. Int. J. Nurs. Stud..

